# Thoracic aortic atherosclerosis in patients with a bicuspid aortic valve; a case–control study

**DOI:** 10.1186/s12872-023-03396-4

**Published:** 2023-07-19

**Authors:** Onur B. Dolmaci, Robert J. M. Klautz, Robert E. Poelmann, Jan H. N. Lindeman, Ralf Sprengers, Lucia Kroft, Nimrat Grewal

**Affiliations:** 1 Department of Cardiothoracic Surgery, Leiden University Medical Center (LUMC), Albinusdreef 2, 2333 ZA Leiden, The Netherlands; 2 Department of Cardiothoracic Surgery, Amsterdam University Medical Center, Amsterdam, The Netherlands; 3 Institute of Biology, Animal Sciences and Health, Leiden University, Leiden, The Netherlands; 4 Department of Vascular Surgery, Leiden University Medical Center (LUMC), Leiden, The Netherlands; 5 Department of Radiology, Amsterdam University Medical Center, Amsterdam, The Netherlands; 6 Department of Radiology, Leiden University Medical Center, Leiden, The Netherlands; 7 Department of Anatomy and Embryology, Leiden University Medical Center, Leiden, The Netherlands

**Keywords:** Bicuspid aortic valve, Aortic wall calcification, Coronary artery disease, Atherosclerosis, Thoracic aortopathy

## Abstract

**Introduction:**

Bicuspid aortic valve (BAV) patients have an increased risk to develop thoracic aortic complications. Little is known about the prevalence and severity of atherosclerosis in the BAV ascending aortic wall. This study evaluates and compares the prevalence of thoracic aortic atherosclerosis in BAV and tricuspid aortic valve (TAV) patients.

**Methods:**

Atherosclerosis was objectified using three diagnostic modalities in two separate BAV patient cohorts (with and without an aortic dilatation). Within the first group, atherosclerosis was graded histopathologically according to the modified AHA classification scheme proposed by Virmani et al. In the second group, the calcific load of the ascending aorta and coronary arteries, coronary angiographies and cardiovascular risk factors were studied. Patients were selected from a surgical database (treated between 2006–2020), resulting in a total of 128 inclusions.

**Results:**

Histopathology showed atherosclerotic lesions to be more prevalent and severe in all TAV as compared to all BAV patients (OR 1.49 (95%CI 1.14 – 1.94); *p* = 0.003). Computed tomography showed no significant differences in ascending aortic wall calcification between all BAV and all TAV patients, although a tendency of lower calcific load in favor of BAV was seen. Coronary calcification was higher in all TAV as compared to all BAV (OR 1.30 (95%CI 1.06 – 1.61); *p* = 0.014).

**Conclusion:**

Ascending aortic atherosclerotic plaques were histologically more pronounced in TAV as compared to the BAV patients, while CT scans revealed equal amounts of calcific depositions within the ascending aortic wall. This study confirms less atherosclerosis in the ascending aortic wall and coronary arteries of BAV patients as compared to TAV patients. These results were not affected by the presence of a thoracic aortic aneurysm.

**Supplementary Information:**

The online version contains supplementary material available at 10.1186/s12872-023-03396-4.

## Background

Atherosclerosis is a systemic disease which can affect the whole arterial system [1]. It has earlier been shown that cardiovascular risk factors such as hypertension, hypercholesterolemia, and diabetes mellitus play an important role in the development of atherosclerotic plaques in the arteries, resulting in cardiovascular diseases with a high morbidity and mortality such as aortic valve stenosis, coronary artery disease and peripheral vascular disease [2–5]. The development of atherosclerosis consists of different stages, starting with endothelial dysfunction and lipid streak formation, progressing to intimal thickening, vascular smooth muscle cell migration and eventually calcific depositions [2]. Computed tomography provides us a way to detect and quantify calcific depositions in patients, and is increasingly used for the diagnosis of coronary artery disease and aortic valve stenosis [6]. Based on these imaging techniques, earlier studies have suggested that thoracic aortopathy might not be associated with systemic atherosclerosis [7]. Plaque calcification is however a late phenomenon in the atherosclerotic disease process. Therefore, (non- and progressive) lesions might go undetected with the imaging techniques used so far to detect aortic atherosclerosis in thoracic aortic aneurysm and dissection patients. To address these earlier stages of atherosclerotic plaques, a histopathological evaluation remains the gold standard.

Patients with a bicuspid aortic valve (BAV) have an increased risk to develop thoracic aortopathy as compared to patients with a tricuspid aortic valve (TAV) [8]. Many studies have till date focused on the pathogenesis leading to the development of an ascending aortic aneurysm and/ or dissection [9–12], in which the role of atherosclerosis remains controversial. Some studies suggest that ascending aortic aneurysm formation itself can be protective for atherosclerosis development, without paying specific attention to the bicuspid population [13]. Studies which have focused on bicuspidy concluded that this population exhibits a lower cardiovascular risk profile and less coronary artery sclerosis as compared to TAV patients [14, 15]. In contrast to these observations, BAV patients have a higher tendency to develop aortic valve stenosis, which is also regarded to be a part of the atherosclerotic disease spectrum, at a younger age and with a higher calcific load and gradient across the aortic valve than TAV patients [15, 16]. Till date, atherosclerosis and calcification of the ascending aortic wall has not been studied specifically in the BAV.

In this study we aim to evaluate the presence and severity of atherosclerosis in the non- and dilated thoracic aortic wall of bicuspid and tricuspid aortic valve patients by using histopathological and imaging modalities. For this purpose we systematically classify atherosclerotic lesions in the ascending aortic wall of BAV and TAV patients according to the adapted AHA classification as proposed by Virmani et al. [17]. Parallelly, in a separate study cohort the calcific depositions in the ascending aortic wall and coronary arteries will be studied as a late stadium of atherosclerosis, results will be compared between the BAV and TAV patients.

## Material and methods

### Study population and ethical approval

Patients with a preoperative CT-scan and coronary angiography who underwent an aortic valve or root replacement between 2006 – 2020, due to an aortic valve stenosis or regurgitation or aortic root enlargement, were included from the Leiden University Medical Center. For the histopathological cohort all consecutive surgically treated BAV and TAV patients between 2013–2014 were included.

Two different cohorts were formed: one cohort (*n* = 69) for the histopathological evaluation of the ascending aorta and another cohort (*n* = 59) for the clinical evaluation (using computed tomography and coronary angiography). Patients under the age of 18, with an aortic dissection or endocarditis were excluded. The medical history of each patient was searched to identify previous coronary artery disease (CAD) events (e.g. myocardial infarction or angina and previous coronary revascularization) and CAD risk factors (i.e. a family history of CAD (aged younger than 65), hypertension, diabetes mellitus, tobacco usage and the body mass index) [18].

Tissue sample collection and handling was carried out according to the official guidelines of the Medical Ethical Committee of Leiden University Medical Center and the code of conduct of the Dutch Federation of Biomedical Scientific Societies (www.FMWV.nl). All patients gave written informed consent.

For the retrospective data analysis the medical ethics committee of the Leiden University Medical Center granted an approval, patient consent was waived.

### Histopathological evaluation

A total of 37 BAV and 32 TAV patients were included for the histopathological evaluation. Aortic wall samples of BAV patients with aortic dilatation (*n* = 19), without aortic dilatation (*n* = 18) and of TAV patients with aortic dilatation (*n* = 14) were obtained from residual aortic wall material during aortic surgery. Samples of TAV patients without aortic dilatation (*n* = 18) were obtained from post-mortem autopsies, which served as a control group. The latter group had a non-cardiac cause of death.

Sample collection was uniform in all patients: ascending aortic wall specimen were obtained from the aortotomy site. The complete circular ascending aortic wall was sectioned in each patient to avoid sampling error of the aortic tissue. Samples were fixed in a 4% formalin solution after excision, decalcified and embedded in paraffin.

Quantitative analysis was performed on samples stained with a MOVAT pentachrome staining. The staining protocol has been described in our earlier work [19, 20]. All samples were analyzed by two researchers independently and atherosclerotic lesions were scored systematically according to the adapted AHA classification as proposed by Virmani et al. [17], which is a classification system in which atherosclerotic lesions are scored based on morphological features (Table 1).

**Table 1:** AHA classification

Morphological description	Associated AHA classification	BAV	TAV	*p*-value
		***n*** ** = 37**	***n*** ** = 32**	
Normal aorta	-	14 (37.8)	4 (12.5)	< 0.001
Nonprogressive intimal lesions		16 (43.2)	12 (37.5)	0.631
- Adaptive intimal thickening	I	9 (24.3)	3 (9.4)	0.105
- Intimal xanthoma	II	7 (18.9)	9 (28.1)	0.37
Progressive atherosclerotic lesions		7 (18.9)	16 (50)	0.01
- Pathological intimal thickening	III	2 (5.4)	4 (12.5)	0.3
- Early fibroatheroma	IV	-	1 (3.1)	0.282
- Late fibroatheroma	IV/V_a_	4 (10.8)	6 (18.8)	0.354
- Thin-cap fibroatheroma	-	1 (2.7)		0.352
- Plaque rupture	VI	-	1 (3.1)	0.282
- Ulcerated plaque	-	-	2 (6.3)	0.126
- Healing rupture	VI	-	-	-
- Healed rupture	-	-	1 (3.1)	0.282
- Fibrotic calcified plaque	V_b,c_, VII	-	1 (3.1)	0.282

### Clinical evaluation and calcific load quantification

The clinical evaluation and comparison of the calcific load in the ascending aortic wall were performed with multidetector row computed tomography (Canon). All patients (*n* = 35 BAV and *n=*24 TAV) had undergone electrocardiogram-triggered computed tomography for calcium scoring. Coronary artery and thoracic aortic wall calcium was scored using the method earlier described by Bijl et al. [21]. Scoring was done independently by two experienced thorax radiologists who were blinded for the clinical patient data. The amount of calcium in the coronary arteries was scored on 8 different anatomical landmarks (the left main, left anterior descending, circumflex artery, right coronary artery and side branches). Furthermore, the thoracic aorta was also studied and calcifications were scored on 4 different anatomical landmarks (the aortic annulus, ascending aorta, arch and proximal descending aorta). The scores ranged between 0 and 2 per anatomical landmark, which eventually were added up and led to a total calcification score for the coronaries (with a maximum score of 16) and the aorta (with a maximum score of 8) for each patient. The amount of calcium was graded as 0 for patients without any calcification, 1 for mild to moderate calcifications and 2 for patients with severe calcifications [21].

Before scanning, metoprolol was administered orally in patients with a resting heart rate of > 65/min in absence of contraindications.

Additionaly, the invasive coronary angiographies of all patients who underwent a computed tomography were studied. The CAGE score was used to score the severity and distribution of coronary obstruction, which scores non-obstructive (CAGE ≥ 20, coronary obstruction of 20–49%) and obstructive (CAGE ≥ 50, coronary obstruction of ≥ 50%) disease in 28 coronary segments (Supplemental Fig. 1, [15, 22–24]). Coronary angiographies were performed by a specialist team within our institution and the results were evaluated by two separate researchers who were blinded for the patient characteristics.

### Statistical analysis

Descriptive continuous data are presented as a mean ± standard deviation or as median and interquartile range depending on the distribution. Categorical data are presented as frequencies and percentages. A normality test (Shapiro–Wilk test), kurtosis and skewness was performed for all variables. Categorical data were compared with a Fisher’s exact test, while continuous variables with normal distribution were compared using T-tests or logistic regression and continuous variables without a normal distribution were compared with the Mann–Whitney U test. An ANOVA analysis was performed to compare means of multiple groups. Only significant variables in univariate analysis were included for correction in a multivariate analysis. Since a protective effect of aortic dilatation on the development of atherosclerosis has been suggested earlier, sub analyses were performed to study the effects of valve morphology and aortopathy separately.A *p*-value < 0.05 was considered statistically significant using SPSS 25.0 (SPSS Inc. Chicago, USA).

## Results

### Histopathological evaluation

A total of 69 patients was included in the histopathology group (Table 2). BAV patients were more often male (78.4% vs 50%, *p* = 0.022) and younger (mean age 58.2 vs 64.8 years, *p* = 0.012) as compared to the TAV individuals. Cardiovascular risk profiles were comparable between BAV and TAV, except for diabetes mellitus which was more prevalent in the TAV group (*p* = 0.045).

**Table 2:** Patient characteristics of histopathology cohort

	**BAV**	**TAV**	**OR (95% CI)**	***p*-value**
N	37	32		
Male	29 (78.4)	16 (50)	3.63 (1.27–10.31)	0.022
Age	58.2 ± 9.1	64.8 ± 10.9	1.07 (1.02–1.12)	0.012
Body Mass Index	25.7 (23.5–29.4)	25.4 (23.1–28.5)	1.01 (0.88–1.15)	0.910
Aortic dilatation^a^	19 (51.4)	14 (43.8)	0.74 (0.29–1.91)	0.631
Hypertension	14 (37.8)	15 (53.6)	1.40 (0.50–3.91)	0.606
Hypercholsterolemia	10 (27)	7 (21.9)	0.70 (0.22–2.19)	0.578
Diabetes mellitus	-	4 (12.5)	0.44 (0.32–0.59)	0.045
Smoking	5 (13.5)	7 (21.9)	1.73 (0.48–6.26)	0.521
Previous CABG	1 (2.7)	2 (6.3)	2.31 (0.20–26.9)	0.599
Previous PCI	1 (2.7)	2 (6.3)	2.31 (0.20–26.9)	0.599

Data are presented as n (%), mean ± SD or median (interquartile range)

*BAV* Bicuspid Aortic Valve, *CABG* Coronary Artery Bypass Grafting, *PCI* Percutaneous Coronary Intervention, *TAV* Tricuspid Aortic Valve

^*a*^*Aortic size of* ≥ *45 mm*

**Fig. 1:**
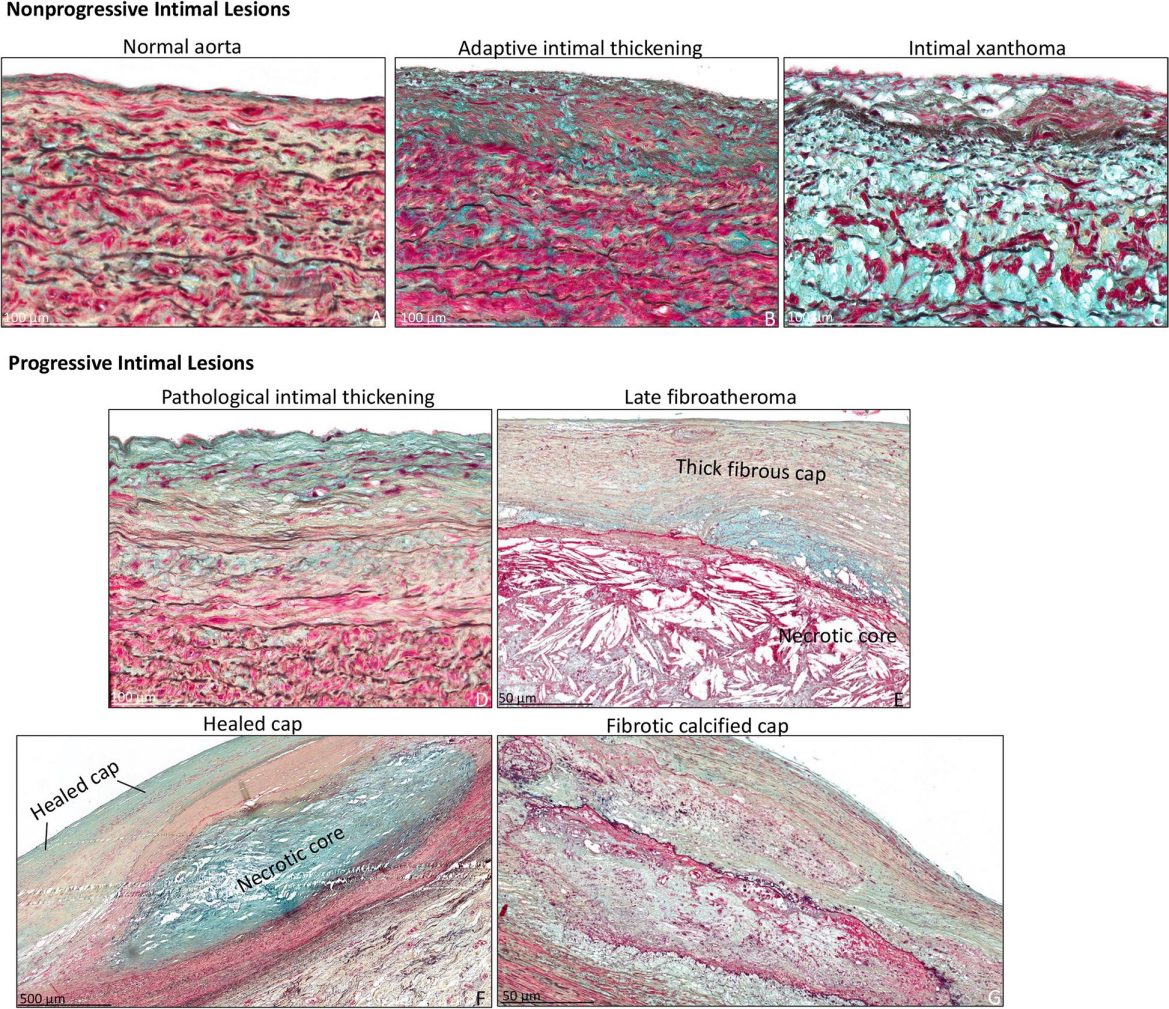
Histopathological evaluation according to the AHA classification. Transverse histologic section of TAA and non-TAA patients (4 μm), stained with MOVAT pentachrome. MOVAT pentachrome stains mucoid extracellular matrix accumulation in light blue, the vascular smooth muscle cells are red, elastic fibers are seen in dark purple, collagen and reticulin in yellow, and nuclei in black. **A**: Normal intimal layer without any signs of adaptive or pathological thinning. **B**: Adaptive thickened intima with mainly vascular smooth muscle cells in the proteoglycan-rich matrix. **C**: Intimal xanthoma with macrophage derived foam cells in the matrix. **D**: Pathological intimal thickening, characterized by the presence of lipid pools deep within the intima near the intimal medial border with overlying vascular smooth muscle cells. **E**: Late fibroatheroma with a necrotic care including cholesterol crystals and a thick fibrous cap. **F**: Healed rupture with a thick and healed fibrous cap. **G**: Fibrotic calcified cap with signs of calcific depositions

The histopathological evaluation and AHA classification [17] are shown in Table 1 and Fig. 1. A total of fourteen BAV patients (37.8%) had a normal aortic wall with no signs of atherosclerosis as compared to four patients (12.5%) in the TAV group (OR 0.42 (95%CI 0.31–0.57); *p* < 0.001). Progressive atherosclerotic lesions were more frequently seen in TAV patients (*n* = 16, 50%) versus BAV (*n* = 7, 18.9%, OR 4.29 (95%CI 1.46–12.57); *p* = 0.010). Evaluation showed atherosclerotic lesions in the ascending aortic wall to be more prevalent and severe in TAV as compared to BAV patients (OR 1.49 (95%CI 1.14 – 1.94); *p* = 0.003) (Fig. 1), which remained significant after correction for the age and sex differences between the two groups (OR 1.38 (95%CI 1.04 – 1.82); *p* = 0.025).

The BAV and TAV patients were further divided into four groups, namely BAV with thoracic aortic aneurysms (BAV-TAA, *n* = 19, 51.4% of all BAVs), BAV non-TAA (*n* = 18, 48.6% of all BAVs), TAV-TAA (*n* = 14, 43.8% of all TAVs) and TAV non-TAA (*n* = 18, 56.3% of all TAVs) to study the effect of aortopathy separately. No difference in prevalence and severity of atherosclerotic lesions was observed between the dilated and non-dilated specimen in both the BAV and TAV. A significant difference in the severity of atherosclerotic lesions was noted between the non-dilated BAV and TAV (*p* = 0.045) and the dilated BAV and TAV (*p* = 0.021).

In addition to the histopathologic evaluation of atherosclerosis according to the AHA classification, the ascending aortic wall was also studied for other pathologic features being elastic fiber thinning, mucoid extracellular matrix accumulation, overall medial degeneration, smooth muscle cell nuclei loss and inflammation, also collectively called the pathology score as described in the consensus statement on surgical pathology of the aorta [25]. This analysis revealed more elastic fiber thinning (*p* > 0.001) and more mucoid extracellular matrix accumulation (*p* < 0.001) in the BAV compared to the TAV patients. TAV patients displayed more overall medial degeneration (*p* < 0.001) and of smooth muscle cell nuclei loss (*p* < 0.001) compared to the BAV. Inflammation tended to be more present in TAV, but did not reach significance in the analysis (*p* = 0.068)*.*

### Clinical evaluation (quantification of calcific load)

**Fig. 2:**
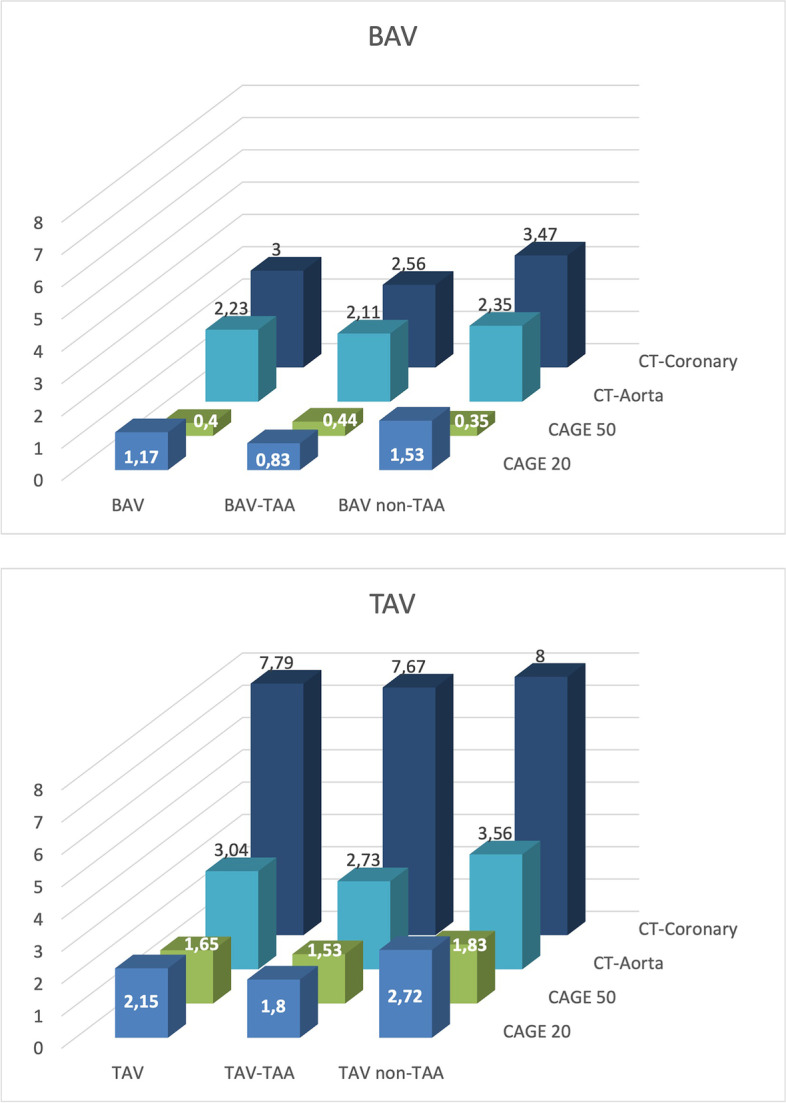
Calcific load. Results of calcific load evaluation using computed tomography and coronary angiography. Results are shown for BAV (upper diagram) and TAV (lower diagram) patients separately. The scores in the diagrams represent the CAGE scores for the coronary angiographies and the calcium score calculated with computed tomography. TAV patients had significantly higher calcific load scores in the coronaries compared to BAV patients (OR 1.25 (95%CI 1.10 – 1.42); *p* = 0.001). Although a tendency of higher calcific scores in the thoracic aortic wall was present in TAV and non-TAA patients, statistical analysis proved both to be non-significant. *BAV* = *Bicuspid Aortic Valve, TAA* = *Thoracic aortic aneurysm, TAV* = *Tricuspid Aortic Valve*

All patients (*n* = 59) were surgically treated due to an aortic valve dysfunction: 37 patients (62.7%) with an aortic valve stenosis and 22 patients (37.3%) with aortic regurgitation. The baseline characteristics of the BAV and TAV groups evaluated with compute tomography and invasive coronary angiography are shown in Table 3, both groups had no differences in age or sex distribution (both *p* > 0.05). Despite having similar cardiovascular risk profiles, TAV patients more often received concomitant coronary bypass grafting (OR 5.54 (95%CI 1.48 – 20.73); *p* = 0.012) compared to BAV patients. Evaluation with computed tomography showed no differences in calcification of the aortic wall between both groups (Fig. 2). Comparisons between patients with an aortic valve stenosis and aortic regurgitation showed only significant differences in the calcific load of the ascending aorta in both BAV (OR 2.50 (95%CI 1.27–4.90); *p* = 0.008) and TAV patients (OR 1.67 (95%CI 1.01–2.76); *p* = 0.044), which was higher in patients with an aortic valve stenosis (see also supplemental Table 1 for patient characteristics).

**Table 3:** Patient characteristics of the clinical cohort (computed tomography)

	**BAV**	**TAV**	**OR (95% CI)**	*p*-value
N	35	24		
Male	29 (82.9)	19 (79.2)	1.27 (0.34–4.76)	0.745
Age	59.9 ± 9.9	62.9 ± 9.9	1.03 (0.98–1.09)	0.251
Indication for surgery				
Aortic valve stenosis	23 (65.7)	14 (58.3)	0.73 (0.25–2.13)	0.594
Aortic regurgitation	12 (34.3)	10 (41.7)	1.37 (0.47–3.99)	0.594
Aortic dilatation^a^	18 (51.4)	15 (62.5)	1.57 (0.55–4.54)	0.436
Aortic size (in mm)	45 (37–51)	52.5 (35–55)	1.03 (0.97–1.09)	0.319
Hypertension	15 (42.9)	14 (58.3)	1.87 (0.65–5.35)	0.295
Hypercholsterolemia	6 (17.1)	8 (33.3)	2.42 (0.71–8.2)	0.214
Diabetes mellitus	3 (8.6)	3 (12.5)	1.52 (0.28–8.28)	0.679
Smoking	5 (14.3)	4 (16.7)	1.29 (0.31–5.44)	0.727
Previous cardiac surgery	2 (5.7)	-	0.58 (0.46–0.72)	0.509
Previous PCI	1 (2.9)	1 (4.2)	1.48 (0.09–24.85)	1.000
Myocardial infarction	2 (5.7)	2 (8.3)	1.5 (0.2–11.45)	1.000
Concomitant CABG	4 (11.4)	10 (41.7)	5.54 (1.48–20.73)	0.012

Data are presented as n (%), mean ± SD or median (interquartile range)

*BAV* Bicuspid Aortic Valve, *BMI* Body Mass Index, *CABG* Coronary Artery Bypass Grafting, *PCI* Percutaneous Coronary Intervention, *TAV* Tricuspid Aortic Valve

^*a*^*Aortic size of* ≥ *45 mm*

Sub analyses were performed by dividing the BAV and TAV patients into four groups, namely BAV-TAA (*n* = 18, 30.5%), BAV non-TAA (*n* = 17, 28.8%), TAV-TAA (*n* = 15, 25.4%) and TAV non-TAA (*n* = 9, 15.3%). Calcific load scores of the aortic wall were the highest for TAV patients without a TAA (3.56) and the lowest in BAV patients with a TAA (2.11), which was not statistically significant (OR 1.58 (95%CI 0.96 – 2.61); *p* = 0.075). Calcific load of the coronary arteries did show differences, being the highest in TAV patients without a TAA (8) and the lowest (2.56) in BAV patients with a TAA (OR 1.30 (95%CI 1.06 – 1.61); *p* = 0.014). Comparison of coronary sclerosis through coronary angiography showed no significant differences between all four groups. Additional analyses were done between TAA and non-TAA patients which showed no significances in cardiovascular risk profiles (Supplemental Table 2).

## Discussion

Atherosclerotic plaques within the non- and dilated ascending aortic wall were histopathologically evaluated in this study of BAV and TAV patients. The lesions were more prevalent and severe in the TAV population. Calcific deposition in the ascending aortic wall and coronary arteries were additionally quantified and compared using imaging modalities in both BAV and TAV patients, which showed a tendency of higher calcific depositions in the aortic wall of TAV patients compared to the BAV, without reaching statistical significance.

BAV patients are at increased risk for developing aortic complications such as aneurysm formation, which might be protective for the development of atherosclerosis [13, 26, 27]. Atherosclerosis is a condition which develops in several stages in which vascular smooth muscle cells play an important role. Vascular smooth muscle cells form a major source of atherosclerotic plaque cells and extracellular matrix at all different stages of atherosclerosis and contribute to numerous processes throughout the disease. Interactions between endothelial cells and vascular smooth muscle cells are important for the development of diffuse and pathological intimal thickening and eventually the development of intimal plaques. Histopathologically, all bicuspid aortic valve patients are characterized by a differentiation defect of the vascular smooth muscle cells [28]. Endothelial cells lining the intimal layer further play a crucial role in the development of the intimal layer by undergoing a transition to mesenchymal cells which is called endothelial to mesenchymal transition [29]. BAV aortas are characterized by an embryonically distorted intimal development, leading to a significantly thinner intimal layer [30]. Vascular wall constituents which are crucial for atherosclerotic plaque development are thus congenitally dysfunctional in the BAV population. Given the high prevalence of bicuspidy in the general population, we hypothesize that the presence of a BAV might be a key factor in the lower prevalence of ascending aortic atherosclerosis in thoracic aneurysm patients seen in earlier studies.

We have previously concluded that the cardiovascular risk profiles and the prevalence of coronary artery disease is not similar between BAV and TAV patients [14, 15], but atherosclerosis in the BAV ascending aortic wall has not been studied yet. Patient tailored risk stratification is of utmost importance in the management of bicuspid aortopathy. In this paper we sought to determine differences in atherosclerosis between the BAV and TAV population as a possible contributing factor for susceptibility for future aortic complications. Our results indicate a tendency of higher calcific load in the aortic wall of TAV patients (Fig. 2), but without reaching statistical significance. The calcific load of the coronary arteries was however significantly higher in the TAV as compared to the BAV patients without resulting in significant coronary obstruction on coronary angiography (p = 0.092). Although the amount of calcification of the thoracic aorta was not significantly different between BAV and TAV patients on computed tomography, histopathological examination did show atherosclerotic lesions to be more prevalent and severe in TAV as compared to BAV patients. Although two separate patient-cohorts were used for the computed tomography and histology studies, our observed differences in results, as expected, confirm a higher sensitivity for detecting atherosclerotic lesions with histology rather than computed tomography. This can most likely be explained by the late occurrence of calcific depositions in the pathophysiology of atherosclerotic lesions, making the earlier stages of plaque formation hard to detect with computed tomography. The latter is in line with our study, in which only 11 (34.4%) of the patients had early signs of calcific depositions.

This study further highlights that the deposition of atherosclerotic plaques and vascular calcification is not homogeneous across the vascular tree, demonstrated by a higher calcific load in the coronaries as compared to the ascending aortic wallwhich also suggests a site of predilection. Coronary arteries, abdominal aorta, iliofemoral arteries and carotid bifurcations are typically affected locations [31]. Side branches, bifurcations and the inner curve of arteries usually have a disturbed (oscillatory) flow and low wall shear stress which makes it preferred locations for plaque formation [32–35]. Wall shear stress has even been shown to play a protective role in plaque formation, possibly due to activation of athero-protective and suppression of pro-atherogenic genes [36, 37].

Based on our findings and the literature we hypothesize that embryonic defects in the development of vascular smooth muscle cells and endothelial cells might be protective for atherosclerosis in the ascending aortic wall of bicuspid aortic valve patients. The effect of differences in wall shear stress across the arterial system, which might explain the predilection for atherosclerosis at certain points across the arterial vascular system has not been evaluated in our study. Future rheologic studies are needed to comprehend whether shear stress can explain differences in the occurrence of atherosclerotic plaques in the coronary arteries as compared to the ascending aorta of patients with an increased cardiovascular risk profile.

### Limitations

The retrospective and single center design carries the known limitations. Furthermore, relatively small patient samples were included, which may be the cause of the moderate significance differences as found within this study. Although corrections were made within the analyses, differences in risk profiles were present between the groups, which may have affected the results.

## Conclusion

Ascending aortic atherosclerotic plaques were histologically more pronounced in all TAV as compared to the BAV patients, while CT scans revealed equal amounts of calcific depositions within the ascending aortic wall. This study confirms less atherosclerosis in the ascending aortic wall and coronary arteries of patients with a BAV as compared to TAV patients. These results were not affected by the presence of a thoracic aortic aneurysm on basis of our subanalysis.

## Supplementary Information

**Additional file 1: Supplemental table 1.** Patient characteristics of BAV and TAV patients in histopathological cohort (divided according to aortic valve disease^†^). **Supplemental table 2.** Patient characteristics of TAA and non-TAA patients in the clinical evaluation cohort (computed tomography). **Supplemental figure 1.** Coronary artery segments (according to CASS) and the corresponding weight factors used for the CAGE score [15, 22–24]. **Supplemental figure 2.** Examples of aortic calcification on computed tomography.

## Data Availability

Data are available upon reasonably request via the corresponding author.

## Abbrevations

BAV: Bicuspid aortic valve
CAD: Coronary artery disease
CT: Computed tomography
TAA: Thoracic aortic aneurysm
TAV: Tricuspid aortic valve
