# Supplementary material for: Thoracic aortic atherosclerosis in patients with a bicuspid aortic valve; a case–control study
Source: BMC Cardiovasc Disord. 2023 Jul 19;23:363. doi: 10.1186/s12872-023-03396-4 (PMC10355074; doi:10.1186/s12872-023-03396-4)
Supplement: Supplementary file 1 — Additional file 1: Supplemental table 1. Patient characteristics of BAV and TAV patients in histopathological cohort (divided according to aortic valve disease†). Supplemental table 2. Patient characteristics of TAA and non-TAA patients in the clinical evaluation cohort (computed tomography). Supplemental figure 1. Coronary artery segments (according to CASS) and the corresponding weight factors used for the CAGE score [15, 22–24]. Supplemental figure 2. Examples of aortic calcification on computed tomography. [file 12872_2023_3396_MOESM1_ESM.docx]

**Supplemental table 1.** Patient characteristics of BAV and TAV patients in histopathological cohort (divided according to aortic valve disease^†^)

|  | **BAV-AS** | **BAV-AR** | **TAV-AS** | **TAV-AR** | **p-value** |
| --- | --- | --- | --- | --- | --- |
| N | 20 | 5 | 6 | 9 |  |
| Male | 13 (65) | 5 (100) | 3 (50) | 4 (44.4) | 0.128 |
| Age | 60.7 ± 10.1 | 53.4 ± 6.5 | 66.2 ± 11 | 63.1 ± 12.8 | 0.105 |
| BMI | 26.3 ± 3.9 | 26.3 ± 2.4 | 30.7 ± 4.0 | 24.6 ± 3.1 |  |
| Aortic dilatation* | 11 (55) | 3 (60) | 2 (33.3) | 9 (100) | 0.668 |
| Hypertension | 8 (40) | 1 (20) | 3 (50) | 3 (66.7) | 0.287 |
| HC | 7 (35) | 1 (20) | 1 (16.7) | 4 (44.4) | 0.680 |
| DM | 0 | 0 | 3 (50) | 0 | 1.000 |
| Smoking | 3 (15) | 1 (20) | 0 | 2 (22.2) | 0.677 |
| Previous CABG | 1 (5) | 0 | 0 | 0 | 0.932 |
| Previous PCI | 1 (5) | 0 | 1 (16.7) | 0 | 0.473 |

* Ascending aortic diameter of ≥ 45mm

† Data reported on patient of whom an echocardiogram was available

AS = Aortic valve stenosis, AR = Aortic regurgitation, BMI = Body Mass Index, CABG = Coronary artery bypass grafting, DM = Diabetes Mellitus, HC = Hypercholesterolemia, PCI = Percutaneous coronary intervention

**Supplemental table 2.** Patient characteristics TAA vs non-TAA patients of the clinical evaluation cohort (computed tomography)

|  | **TAA** | **Non-TAA** | **OR (95% CI)** | **p-value** |
| --- | --- | --- | --- | --- |
| N | 33 | 26 |  |  |
| Age | 62.7 ± 9 | 59.2 ± 10.9 | 1.04 (0.98-1.1) | 0.182 |
| Male | 27 (81.8) | 21 (80.8) | 0.93 (0.25-3.48) | 1.000 |
| Indication for surgery  Aortic valve stenosis  Aortic regurgitation | 16 (48.5)  17 (51.5) | 21 (80.8)  5 (19.2) | 0.22 (0.07-0.74)  4.46 (1.36-14.68) | 0.015  0.015 |
| Bicuspid Aortic Valve | 18 (54.5) | 17 (65.4) | 1.57 (0.55-4.54) | 0.436 |
| Aortic size (in mm) | 54 (50-55) | 36.5 (32.5-39.5) | 1.66 (1.24-2.23) | 0.001 |
| Hypertension | 18 (54.5) | 11 (42.3) | 1.64 (0.58-4.62) | 0.435 |
| Hypercholsterolemia | 5 (15.2) | 9 (34.6) | 0.34 (0.1-1.18) | 0.123 |
| Diabetes mellitus | 2 (6.1) | 4 (15.4) | 0.36 (0.06-2.11) | 0.390 |
| Smoking | 7 (21.2) | 2 (7.7) | 3.65 (0.69-19.45) | 0.154 |
| Previous cardiac surgery | - | 2 (7.7) | 0.42 (0.31-0.57) | 0.190 |
| Previous PCI | - | 2 (7.7) | 0.42 (0.31-0.57) | 0.190 |
| Myocardial infarction | 3 (9.1) | 1 (3.8) | 2.5 (0.25-25.56) | 0.623 |
| Concomitant CABG | 8 (24.2) | 6 (23.1) | 1.07 (0.32-3.58) | 1.000 |

Data are presented as n (%), mean ± SD or median (interquartile range).

*BMI = Body Mass Index, CABG = Coronary Artery Bypass Grafting, PCI = Percutaneous Coronary Intervention, TAA = Thoracic Aortic Aneurysm*

**Supplemental figure 1.** Coronary artery segments (according to CASS) and the corresponding weight factors used for the CAGE score [15, 22-24].

**
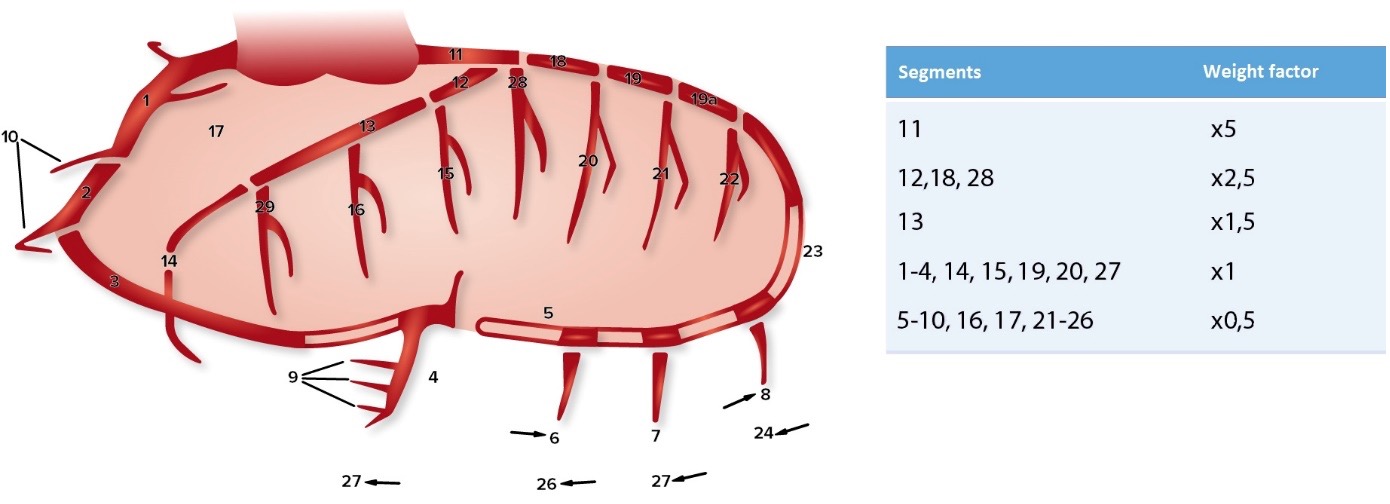
**

**Supplemental figure 2. Examples of aortic calcification on thoracic computed tomography sections**

**
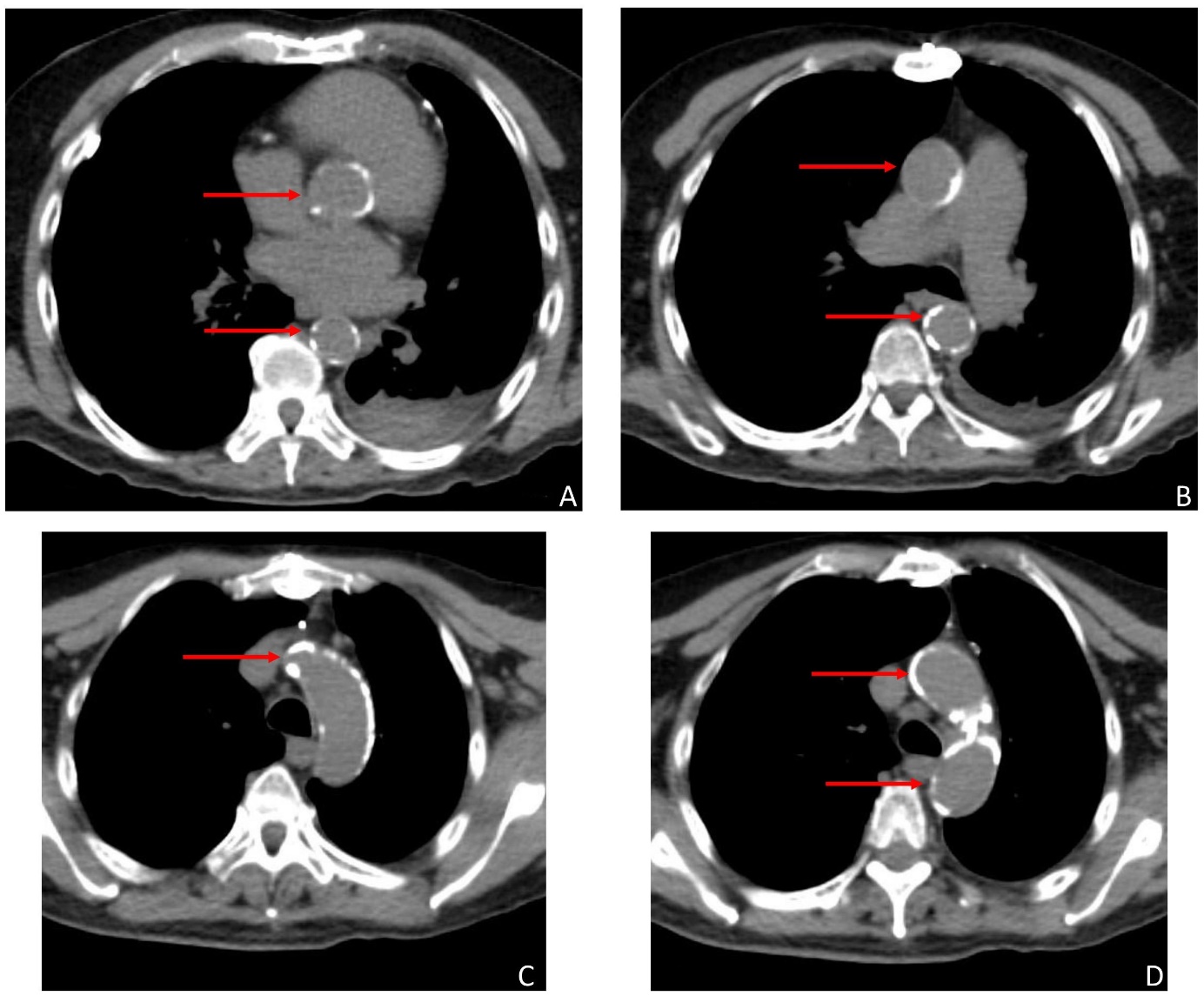
**

Transverse sections of a thoracic computed tomography scan are shown in A-D. In this study aortic calcifications were scored on four different anatomical landmarks being the aortic annulus, ascending aorta, arch and proximal descending aorta. Figure A: Example of severe calcifications in the aortic annulus and descending aorta, indicated with a red arrow. Figure B: Example of severe calcifications in the ascending aorta and descending aorta, indicated with a red arrow. Figure C: Example of severe calcifications in the aortic arch, indicated with a red arrow. Figure D: Severe calcifications in the aortic arch and proximal descending aorta.
